# Age‐related differences in brain network activation and co‐activation during multiple object tracking

**DOI:** 10.1002/brb3.533

**Published:** 2016-09-07

**Authors:** Erlend S. Dørum, Dag Alnæs, Tobias Kaufmann, Geneviève Richard, Martina J. Lund, Siren Tønnesen, Markus H. Sneve, Nina C. Mathiesen, Øyvind G. Rustan, Øivind Gjertsen, Sigurd Vatn, Brynjar Fure, Ole A. Andreassen, Jan Egil Nordvik, Lars T. Westlye

**Affiliations:** ^1^ Sunnaas Rehabilitation Hospital HTNesoddenNorway; ^2^ NORMENTKG Jebsen Centre for Psychosis ResearchDivision of Mental Health and AddictionOslo University Hospital & Institute of Clinical MedicineUniversity of OsloOsloNorway; ^3^ Department of PsychologyUniversity of OsloOsloNorway; ^4^ Department of RadiologyOslo University HospitalOsloNorway; ^5^ Department of Geriatric MedicineOslo University HospitalOsloNorway

**Keywords:** attention, cognitive aging, DAN, default mode network, DMN, dorsal attention network, MOT, multiple object tracking

## Abstract

**Introduction:**

Multiple object tracking (MOT) is a powerful paradigm for measuring sustained attention. Although previous fMRI studies have delineated the brain activation patterns associated with tracking and documented reduced tracking performance in aging, age‐related effects on brain activation during MOT have not been characterized. In particular, it is unclear if the task‐related activation of different brain networks is correlated, and also if this coordination between activations within brain networks shows differential effects of age.

**Methods:**

We obtained fMRI data during MOT at two load conditions from a group of younger (*n* = 25, mean age = 24.4 ± 5.1 years) and older (*n* = 21, mean age = 64.7 ± 7.4 years) healthy adults. Using a combination of voxel‐wise and independent component analysis, we investigated age‐related differences in the brain network activation. In order to explore to which degree activation of the various brain networks reflect unique and common mechanisms, we assessed the correlations between the brain networks' activations.

**Results:**

Behavioral performance revealed an age‐related reduction in MOT accuracy. Voxel and brain network level analyses converged on decreased load‐dependent activations of the dorsal attention network (DAN) and decreased load‐dependent deactivations of the default mode networks (DMN) in the old group. Lastly, we found stronger correlations in the task‐related activations within DAN and within DMN components for younger adults, and stronger correlations between DAN and DMN components for older adults.

**Conclusion:**

Using MOT as means for measuring attentional performance, we have demonstrated an age‐related attentional decline. Network‐level analysis revealed age‐related alterations in network recruitment consisting of diminished activations of DAN and diminished deactivations of DMN in older relative to younger adults. We found stronger correlations within DMN and within DAN components for younger adults and stronger correlations between DAN and DMN components for older adults, indicating age‐related alterations in the coordinated network‐level activation during attentional processing.

## 1 Introduction

Attention entails a differential allocation of cognitive resources toward task‐relevant information at the expense of that deemed less relevant. It is a core property of perceptual and cognitive operations, and in common with a range of other cognitive domains, declines with age throughout the adult lifespan (McDowd & Shaw, 2000). Although the mechanisms and neural correlates of such age‐related decline are not completely understood, the most consistent findings to date point toward disruptions in interconnected brain networks (Andrews‐Hanna et al., 2007; Parks & Madden, 2013).

Studies in functional neuroimaging have identified neural networks with differential responses during task paradigms. Networks routinely exhibiting increased activity during tasks are described as task‐positive networks (TPN) (Duncan, 2013; Hugdahl, Raichle, Mitra, & Specht, 2015), while another well‐described network showing an opposite pattern of activation to that of TPN, is the default mode network (DMN). Typically, DMN exhibits higher activity during resting conditions and decreased activation during cognitively demanding tasks (Beckmann, DeLuca, Devlin, & Smith, 2005; Raichle et al., 2001). A dorsal frontoparietal TPN known as the dorsal attention network (DAN) has been proposed as the source of endogenous, top‐down attention signals in the brain (Ruff et al., 2008). DAN is involved in mapping task‐relevant sensory information to adequate behaviors, and supports sustained and selective visuospatial attention through biasing the competition for representational space in sensory cortices (Corbetta, Kincade, Ollinger, McAvoy, & Shulman, 2000; Corbetta, Patel, & Shulman, 2008; Corbetta & Shulman, 2002; Gitelman et al., 1999), and by increasing the baseline activity for an attended object, suppressing distractors and limiting the number of object representations (Bar, 2003; Pessoa, Kastner, & Ungerleider, 2003). Critical nodes include the superior parietal lobe (SPL), inferior parietal sulcus (IPS), posterior parietal cortex, and the frontal eye fields (FEF) (Fox et al., 2005; Szczepanski, Pinsk, Douglas, Kastner, & Saalmann, 2013; Toro, Fox, & Paus, 2008). A recent meta‐analysis by Li et al. (2015) reviewing age‐related changes in activations during tasks encompassing multiple cognitive domains including attention, memory, and executive function, indicated a crucial role of increased DAN in successful compensation for older adults. Insight into the effects of aging on DAN modulation is therefore an attractive target for studies aiming to delineate the neural mechanisms of cognitive aging.

While the DAN and other TPNs display increased activation in response to demanding tasks, the DMN, including the medial prefrontal and medial parietal cortex, the posterior cingulate and precuneus, and the medial temporal lobe (Spreng, Mar, & Kim, 2009; Uddin, Clare Kelly, Biswal, Xavier Castellanos, & Milham, 2009), typically shows increasing activity during periods without specific task demands (Raichle et al., 2001). The dichotomous relationship between these networks generally becomes more pronounced as attentional demand increases, reflected both in increased activation in DAN (Wojciulik & Kanwisher, 1999) and increased deactivation in DMN (Mckiernan, Kaufman, Kucera‐Thompson, & Binder, 2003).

Some studies suggest that the enhancement of relevant information largely remains intact, while efficient neural suppression of irrelevant information seems to be compromised in aging (Alain & Woods, 1999; Czigler, Csibra, & Ambró, 1996; Gazzaley, Cooney, Rissman, & D'Esposito, 2005). Other reported age‐related decline both in task‐related enhancement as well as inefficient suppression (Logan, Sanders, Snyder, Morris, & Buckner, 2002; Tomasi, Wang, Wang, & Volkow, 2014). Previous fMRI studies have shown less pronounced DMN deactivation during cognitive tasks (Damoiseaux et al., 2008; Grady, Springer, Hongwanishkul, McIntosh, & Winocur, 2006; Lustig et al., 2003; Miller et al., 2008; Persson, Lustig, Nelson, & Reuter‐Lorenz, 2007) as well as reduced resting‐state DMN connectivity (Esposito et al., 2008; Mowinckel, Espeseth, & Westlye, 2012) in older adults. Diminished DMN deactivations during task performance may indicate reduced network regulation and dynamic range of network modulation to altering task demands (Spreng & Schacter, 2012), supporting the view of the aging brain as more rigid and less cortically selective within the given cognitive states. In line with the hypothesis that disruptions of large‐scale brain networks contribute to aging‐related cognitive decline, Andrews‐Hanna et al. (2007) observed age‐related decreases in functional connectivity within both DMN and DAN, which were associated with poor cognitive performance.

In this study, we investigated age differences in the task‐related activations across a range of brain networks during multiple object tracking (MOT) (Pylyshyn & Storm, 1988). MOT is a powerful paradigm for measuring sustained goal‐driven attention, requiring participants to attend multiple target items as they move among distractors. Attentional load can be manipulated by increasing the number of objects the subject is requested to track; higher numbers of objects to track implies higher attentional load. Load manipulation allows for distinguishing between brain regions directly involved in attentional performance and showing load‐dependent activity from regions activated by the task alone, giving a better measure for estimating neural activity in response to attentional demand rather than activity produced by task‐relevant, but not load‐dependent functions such as suppression of eye movement (Culham, Cavanagh, & Kanwisher, 2001). Tracking performance is reduced with age for trials with higher attentional load (Trick, Perl, & Sethi, 2005), and this reduction in performance may be specific to attentional function, as memory for object location was only marginally affected by age (Sekuler, McLaughlin & Yotsumoto, 2008). A recent event‐related potentials (ERP) study reported age‐related decreases in tracking performance with reduced attentional modulation of the visual P1 component (Störmer, Li, Heekeren, & Lindenberger, 2013; Trick et al., 2005), suggesting that MOT is sensitive to age‐related differences in the neuronal machinery supporting attention.

While fMRI studies have documented robust DAN activation during MOT (Culham et al., 1998, 2001; Howe, Horowitz, Morocz, Wolfe, & Livingstone, 2009; Jovicich et al., 2001), age‐related differences have not been studied. Further, little is known about DMN modulation during tracking (but see Alnæs et al., 2015; Tomasi et al., 2014), and to which degree network‐specific activation and deactivations of different brain networks reflect independent and overlapping predictors of cognitive aging. Using independent component analysis (ICA) and conventional voxel‐wise time‐series analyses, our main aims were to test for differences between a group of younger and older healthy volunteers in brain network activation and deactivation across a range of brain networks, including networks involved in motor, sensory, and cognitive functions, during MOT. Secondly, in order to explore to which degree activation of the various brain networks reflect unique and common mechanisms, we assessed the correlations between the brain networks' activations.

Based on current models of cognitive and brain aging (Cabeza, Anderson, Locantore, & McIntosh, 2002; Damoiseaux et al., 2008; Dennis & Cabeza, 2008; Grady et al., 2006; Li et al., 2015; Reuter‐Lorenz & Park, 2010), we hypothesized:

1. MOT engages a range of brain networks, including but not limited to the DAN and DMN. Network responses are more pronounced with increasing load demand and there is an interaction effect of load and age, with stronger age‐related differences at higher load levels.
2. Age differences are particularly manifested as reduced DMN deactivation and altered DAN activation, partly reflecting reduced tracking performance (Nagel et al., 2009). DAN effects may manifest either as increased activation in the old group, which—when accompanied by similar performance—indicate some form of compensation or increased mental effort associated with task demands (Cabeza et al., 2002; Reuter‐Lorenz & Lustig, 2005), or as reduced activation, reflecting diminished brain network efficiency.
3. Based on the concept that cognition is enabled by the temporal synchronization of different brain networks and in line with the notion of dedifferentiation in cognitive aging (Andrews‐Hanna et al., 2007; Baltes & Lindenberger, 1997; Chan, Park, Savalia, Petersen, & Wig, 2014; Lindenberger, 2014), we anticipated that age effects would additionally be revealed in a differential pattern of correlations in levels of task‐related activations between the two age groups. The dedifferentiation theory posits that aging confers a loss of network specificity and disruptions within intrinsic functional networks, particularly implicating DMN and DAN (Andrews‐Hanna et al., 2007). Thus, we anticipate stronger correlation in levels of co‐activation between task‐related components, particularly within the DAN and DMN for the younger group compared to the older group.

## 2 Materials and Methods

### 2.1 Sample

We recruited 26 young (mean age: 24.2 years, *SD*: 4.9, 69% females) and 26 old (mean age: 66.2 years, *SD*: 7.4, 58% females) adults through a newspaper ad and social media. All subjects underwent neuropsychological screening (details below). Participants reported normal or corrected‐to‐normal vision. Exclusion criteria included estimated IQ < 70, previous history of alcohol‐ and substance abuse, history of neurologic or psychiatric disease, participants presently on any medication significantly affecting the nervous system and counterindications for MRI. All participants were self‐sufficient and living independently, and reported no reason to suspect marked cognitive decline or undiagnosed dementia.

From the full dataset, we excluded participants due to excess motion during scanning (*n* = 2) as well as poor MOT performance (*n* = 5). Specifically, participants performing worse than chance during the low load condition (see below) or 2.5 standard deviations below the mean during the high load condition were excluded from analyses. Outliers in the high load condition were determined by boxplot graph visualization and verified using Grubbs' test for single outlier (Iglewicz & Hoaglin, 1993), yielding a final sample of 24 younger (mean age 24.42 years, *SD*: 5.06, 66.7% females) and 21 older adults (mean age 64.67, *SD*: 7.44, 53.4% females).

### 2.2 Screening and neuropsychological assessment

Participants completed the matrices and vocabulary subtests from the Wechsler Abbreviated Scale of Intelligence (WASI; Wechsler, 1999) as a measure of general intellectual functioning, and the 4‐trial version of the Stroop Color Word Interference test (CWIT) from the Delis–Kaplan Executive Function System (D‐KEFS; Delis, Kaplan, & Kramer, 2001) to obtain a measure of cognitive speed, interference, and inhibition. CWIT comprises the following four conditions: (1) Color task—subject identifies the color of a series of squares (red, blue, green); (2) Word task—subject reads a series of color words (red, blue, green) written in black print; (3) Inhibition task—subject is presented with words written in congruent (e.g., red written in red ink) and incongruent (e.g., red written in blue ink) colors; (4) Inhibition/Switching—subject is presented with words written in congruent and incongruent colors. We used completion times as basis for analysis, with focus on the inhibition and inhibition/switching tasks.

Wechsler Abbreviated Scale of Intelligence subtest scores were converted to standardized scores from which full‐scale IQ (FSIQ) was estimated. Two subjects (one young and one old) were native foreign language speakers and thus were not adequately able to perform the vocabulary subtest; their scores on this subtest were reported as missing and their FSIQ was calculated solely based on performance in the matrix reasoning subtest.

### 2.3 MOT paradigm

All participants performed two versions of MOT in the MRI scanner during the same session, including one blocked run, and two runs comprising continuous tracking, in addition to one resting‐state run following the protocol used by Alnæs et al. (2015). Here, we report data from the blocked runs. The level of attentional demand was set at two load conditions—load 1 (L1) and load 2 (L2) requiring the participants to track one or two targets, respectively, during the task. We restricted the load level to a maximum of L2 to ensure that both group were able to perform at a high level, which is in particular pertinent for the subsequent utilization of the paradigm in clinical populations.

For both versions, the participant was looking through a mirror mounted on the head coil at a MR‐compatible LCD screen (NNL LCD Monitor^®^, NordicNeuroLab, Bergen, Norway) placed in front of the scanner bore, with a screen resolution of 1920 × 1080@60 Hz. All stimuli were generated using MATLAB and the Psychophysics Toolbox extensions (Brainard, 1997; Pelli, 1997). Participants produced their responses using a MR‐compatible subject response collection system (ResponseGrip^®^, NordicNeuroLab). A trigger pulse from the scanner synchronized the onset of the experiment to the beginning of the acquisition of an fMRI volume. The screen covered 32.43° of visual angle, and the tracking area covered 17.32° of visual angle at a viewing distance of 1.2 meters. The objects were circular disks with a diameter of 0.7° of visual angle moving at a speed of 4°/second. Objects changed direction when closer than 1° to another object or the edge of the tracking area, and also made random changes (one random turn per second on average) to make object movements unpredictable. A fixation circle of 0.5° of visual angle was located in the center of the tracking area. The participant's task consisted of covertly tracking target objects while fixating on the central fixation point. Detailed instructions were given before entering the scanner room as well as before each sequence.

The blocked run contained 18 trials divided into six blocks. Each block contained three different conditions: Passive Viewing (PV), one target object (L1), and two target objects (L2), followed by rest. The rest periods, which lasted 12 s were not explicitly modeled and thus constitute the implicit baseline. The order of the three conditions was random and counterbalanced so that each condition was followed by a rest period in two of the six parts, that is, PV was presented twice followed by rest, and so were L1 and L2. The run started with 1.5 s of instructions followed by 0.5 s fixation. Then, 10 identical objects were presented on a gray background. All objects were blue for 0.5 s, and then either zero (PV condition), one (L1) or two (L2) of the objects turned red (designating them as targets) for 2.5 s before turning color back to blue (during tracking all objects were identical). After another 1.5 s, the objects started moving randomly and independently of each other for 12 s. At the end of each trial, the objects stopped moving before one of the objects turned green (probe) for 2.5 s. The participant was instructed to respond as quickly and accurately as possible, “yes” or “no” to whether the green probe was one of the objects originally designated as a target. In passive viewing trials, there were no targets, nor probe, but the participants were still instructed to keep fixation during the length of the trial. Accuracy and response time (RT) were recorded for each button press.

### 2.4 MRI acquisition

Magnetic resonance imaging scans were obtained from a General Electric (Signa HDxt) 3.0T scanner with an 8‐channel head coil at Oslo University Hospital. Functional data were acquired with a T2*‐weighted 2D gradient echo planar imaging sequence (EPI) with 217 volumes (TR: 2,400 ms; TE: 30 ms; FA: 90°; voxel size: 3.75 × 3.75 × 3.2 mm; slices: 48; FOV: 240 × 240 mm; duration: 533 s). The first five volumes were discarded to allow for T1 equilibrium. In addition, a structural scan was acquired using a sagittal T1‐weighted fast spoiled gradient echo (FSPGR) sequence (TR: 7.8 s; TE: 2.956 ms; TI: 450 ms; FA: 12°; voxel size: 1.0 × 1.0 × 1.2 mm; slices: 170; FOV: 256 mm²; duration: 428 s).

### 2.5 fMRI processing and analysis

Functional MRI data were processed on single‐subject level using the FMRI Expert Analysis Tool (FEAT) from the FMRIB Software Library (FSL; Smith et al., 2004), including spatial smoothing (FWHM = 6 mm), high‐pass filtering (sigma = 64 s), motion correction (MCFLIRT), and single‐session ICA using MELODIC (Beckmann & Smith, 2004).

We calculated in‐scanner subject motion defined as the average root mean square of the displacement from one frame to its previous frame for each dataset, and used FMRIB's ICA‐based Xnoiseifier (FIX; Salimi‐Khorshidi et al., 2014) to identify and remove noise components (standard training set, threshold: 20), yielding a cleaned dataset for each subject. We used 24 motion parameters, including 6 raw realignment parameters and 24 extended parameters estimated from the realignment procedure. We did not regress out the global signal (GSR), nor the white matter or CSF. Instead, we used an ICA‐based approach to selectively regress out noise components (FIX) from each dataset, in line with recent studies evaluating benefits of different noise reduction strategies (Pruim, Mennes, Buitelaar, & Beckmann, 2015). The older group showed significantly more in‐scanner motion, *t*(43) = −3.9, *p* < .0001, and FIX removed a significantly higher number of components from the older group, *t*(19) = −2.2, *p* < .05, and also removed more of the variance from the raw fMRI data, both in terms of absolute and relative variance, both *t*(19) = −2.8, *p* < .05), which were significantly correlated with amount of subject motion across groups (*r* = .52, *p* < .0001).

We used FreeSurfer (Fischl et al., 2002) for automated brain segmentation of the T1‐weighted data to obtain brain masks used for co‐registration to a standard coordinate system using FLIRT (Jenkinson & Smith, 2001), optimized using boundary‐based registration (BBR; Greve & Fischl, 2009) and FNIRT (Andersson, Jenkinson, & Smith, 2007a,b).

### 2.6 Voxel‐wise GLMs

In the first‐level general linear model (GLM), the onset and duration of the PV and tracking blocks (L1 and L2) were modeled with the fixation blocks as implicit baseline. The design matrix was filtered and convolved with a hemodynamic response function (HRF) before the model fit. A temporal derivative was added to the model to adjust for regional differences in the timing of the HRF. We included the following contrasts: Tracking (average of L1 and L2) versus PV, and L1 versus L2. The individual contrast parameter maps were then subjected to whole‐brain group analysis based on a random effects model, testing for differences between the young and the old group. To correct for multiple comparisons across space, we performed cluster‐level correction with voxel‐wise Z > 2.3 and a corrected cluster significance threshold of *p* < .05 for all analyses.

### 2.7 Group‐independent component analysis and time‐series analysis

The individually processed, filtered, cleaned, and normalized fMRI volumes were submitted to a group‐level ICA using the temporal concatenation approach in MELODIC (Beckmann & Smith, 2004). The number of components was calculated using a Laplace approximation of the posterior probability of the model order (Beckmann & Smith, 2004), yielding 41 components. Next, the group‐average spatial maps for the 28 non‐noise components were used to generate subject‐specific maps and associated time series using dual regression (Filippini et al., 2009). Dual‐regression time series were submitted to time‐series regression using the same individual‐level GLM design matrices used for the voxel‐wise analysis. The regression coefficients for the two tracking conditions were subtracted for each participant (L2‐L1) for both DMN and DAN and submitted to group‐level analysis assessing main effects of load across groups, as well as differences between the old and the young group. For visualization purposes, we calculated the group‐average blocked time series for each of the three MOT conditions (PV, L1, L2) based on the z‐normalized (within‐run) dual‐regression time series for each subject.

### 2.8 Statistical analysis

Nonimaging data were analyzed in SPSS (IBM_Corp, 2010). Between‐group differences were assessed using Chi square tests (sex distribution) and linear models (age, neuropsychological performance, task performance). Group differences in MOT performance and estimated brain network beta values were assessed using two‐by‐two repeated‐measures ANOVA with load (L1 and L2) as within subject factor and group (young and old) as between subject factor. Group differences in beta values were further explored using ANCOVA including gender and group as fixed factors. We used paired samples *t*‐tests and ANCOVA to test for load‐dependent (L2‐L1) differences in beta values. To test for association between task performance and beta estimates, we used an ANCOVA with gender and group as fixed factors and task accuracy during L2 as the dependent variable. We tested for association between neuropsychological performance and beta estimates using an ANCOVA with gender and group as fixed factors and performance scores for each neuropsychological subtest as the dependent variable. Lastly, we employed an ANCOVA to test for associations between the beta estimates for the respective networks at the given load conditions for the two groups, covarying for gender and age. In order to assess the between‐network correlations of the task‐related activations, we computed the Pearson's correlation between the betas of each of the component pairs, yielding a 28 by 28 correlation matrix for each group. Next, in order to test for group differences, the correlation coefficients were compared between groups using Fisher's r‐to‐z transformation. The resulting *p*‐values were then adjusted using Bonferroni and FDR corrections. Raw *p*‐values are also shown for transparency.

## 3 Results

### 3.1 Demographics, neuropsychology, and task performance

Table 1 summarizes demographic, neuropsychological, and MOT performance. Briefly, the younger subgroup performed significantly better on the matrix reasoning task of the WASI subtest as well as the inhibition and inhibition/switching parts of the Stroop test. No other significant differences were found. An ANCOVA exploring whether differences in performance on the neuropsychological tests correlated with activation levels in the selected DAN and DMN components revealed no significant correlation between WASI matrix reasoning and beta coefficients for either group. ANCOVA associating completion time during the inhibition task from the Stroop task with activation levels for DAN L2 revealed a main effect of group [*F*(1,40) = 4.94, *p* = .032, partial η^2 ^= .110], but no main effect of DAN L2 [*F*(1,40) = .88, *p* = .354, partial η^2 ^= .021] and no interaction effect [*F*(1,40) = 3.88, *p* = .056, partial η^2 ^= .088]. No significant association was found between DMN L2 and performance on the inhibition subtest. ANCOVA associating completion time during the inhibition/switching task from the Stroop test with activation levels for DAN L2 revealed a main effect of group [*F*(1,40) = 5.88, *p* = .020, partial η^2 ^= .128], a main effect of DAN L2 [*F*(1,40) = 7.46, *p* = .009, partial η^2 ^= .009], and an interaction effect [*F*(1,40) = 5.42, *p* = .025, partial η^2 ^= .119], indicating better performance with increased DAN activation for the young group. Association between DMN L2 and the inhibition/switching subtest revealed no main effect of group [*F*(1,40) = .01, *p* = .930, partial η^2 ^= .000], but a main effect of DMN L2 [*F*(1,40) = 5.23, *p* = .027, partial η^2 ^= .116] and an interaction effect [*F*(1,40) = 4.94, *p* = .032, partial η^2 ^= .110], indicating better performance with increased DMN deactivation for the young group.

**Table 1:** Demographics and neuropsychological performance

	Young (*SD*)	Old (*SD*)	χ^2^/*t*‐score	*p*
*N*	24	21		
Age	24.42 (5.06)	64.67 (7.44)		
Age range	20–43	47–78		
Percent male	33.3	47.6	χ^2 ^= 0.95	.33
Percent right handedness	91.7	85.7	χ^2 ^= 1.61	.21
Years of education	15.50 (1.37)	15.12 (3.06)	*t* = 0.53	.603
WASI matrix reasoning	29.58 (2.17)	25.71 (6.48)	*t* = 2.61	**.015**
WASI vocabulary	65.91 (6.20)	66.10 (10.6)	*t* = −0.07	.943
Full scale IQ‐2	120.04 (9.17)	119.95 (17.45)	*t* = 0.02	.983
Stroop word	30.36 (6.86)	34.36 (7.39)	*t* = −1.83	.074
Stroop color	22.79 (5.27)	25.33 (5.30)	*t* = −1.61	.115
Stroop inhibition	47.17 (11.26)	64.14 (22.35)	*t* = −3.15	**.004**
Stroop inhibition/switching	55.50 (11.73)	79.19 (45.94)	*t* = −2.30	**.031**
MOT accuracy on L1	97.9 (7.47)	94.4 (10.9)	*t* = 1.22	.230
MOT accuracy on L2	88.9 (14.5)	77.8 (20.6)	*t* = 2.063	**.047**

Significant group differences are shown in bold.

Mean tracking accuracy during the MOT task was 97.9% (L1) and 88.9% (L2) and 94.4% (L1) and 77.8% (L2) for the young and old group, respectively. A two‐by‐two repeated‐measures ANOVA with load (L1 and L2) as within subject factor and group (young and old) as between subject factor yielded a significant effect of load [*F*(1,43) = 26.04, *p* = 7.0E‐6] and group [*F*(1,43) = 4.69, *p* = .04], but no load by group interaction [*F*(1,43) = 2.30, *p* = .137].

### 3.2 Functional MRI

#### 3.2.1 Voxel‐wise analysis

**Figure 1:**
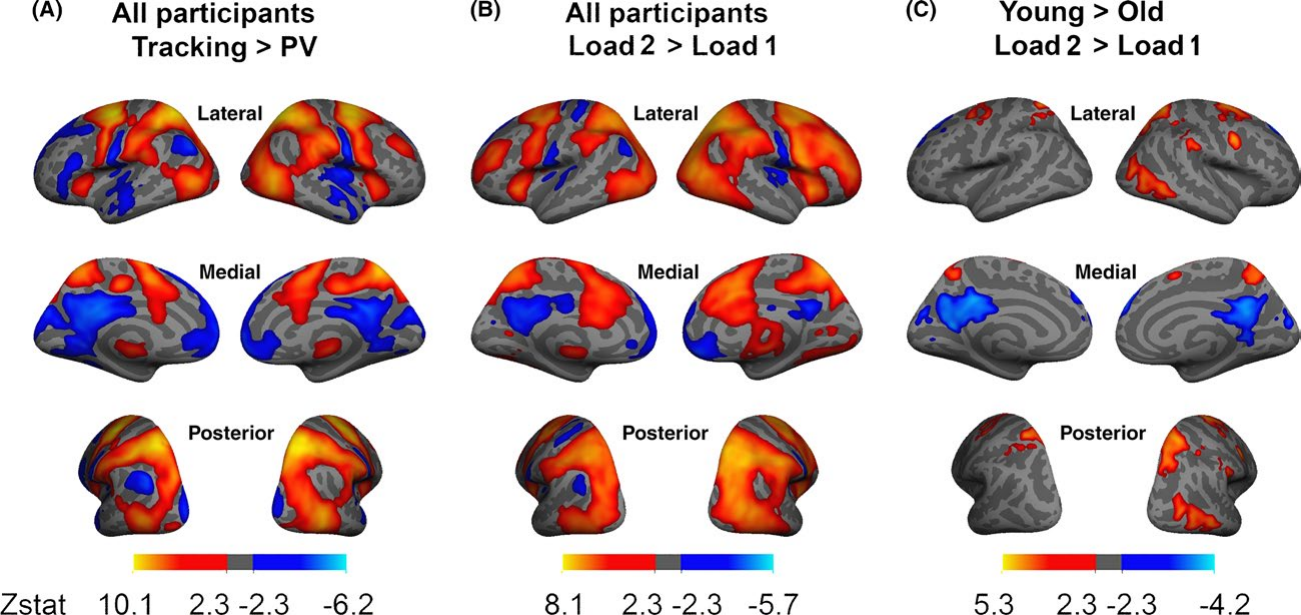
Voxel‐wise GLM analysis for the contrasts; (A) Tracking > passive viewing across groups; (B) L2 > L1 across groups; (C) Young > old group for L2 > L1. We employed Gaussian random field theory to carry out cluster‐level corrections for multiple comparisons (voxel‐level *Z* > 2.3; cluster significance: *p* < .05, corrected)

In order to validate the MOT paradigm, we investigated whether patterns of activation in this study mirrored previously described findings in MOT research. Figure 1A shows the main effects across groups for the tracking versus PV contrast. Tracking‐related activation is seen in human motion area (MT+), FEF, IPS, SPL, and SMA. Tracking‐related deactivation is seen within the DMN including the medial prefrontal cortex (mPFC), the precuneus, and the lateral temporal cortex (LTC).

Figure 1B shows the main effects across groups for the L2 versus L1 contrast. Increasing the task demand produces load‐related activations and deactivations in areas overlapping the tracking versus PV contrast, including increased activations in FEF, lateral occipital cortices, and superior parietal lobules, decreases in activations are seen in medial prefrontal cortices and precuneus.

Figure 1C and Table 2 summarize the significant group differences in the L2 versus L1 contrast. Four clusters showed significantly greater load‐dependent activity increases in the young compared to the old group, including the FEF, MT+ and SPL. Two clusters showed greater load‐related deactivations in the young compared to the old group, including the precuneus and the mPFC.

**Table 2:** Cluster list with coordinates, cluster‐level statistics for local maxima and associated brain regions for the main effects of the L2 > L1, young > old contrast. Positive *Z*‐scores reflect increased differentiation between L2 and L1 in the young compared to the old group

Brain region	Voxels	*p*	−log10(*p*)	*Z*‐max	*Z*‐Max *x*,* y*,* z* (mm)
Right SPL	4,879	1.5E‐08	7.81	5.03	62, −46, 52
Right FEF	2,285	8.66E‐05	4.06	5.27	20, −6, 78
Left FEF	1,133	0.0115	1.94	4.2	−32, −4, 60
Right MT+	1,014	0.0206	1.69	4.48	40, −72, 6
Left precuneus	4,564	5.96E‐08	7.22	−4.2	−6, −38, 34
Left mPFC	2,671	2.06E‐05	4.69	−4.02	16, 42, 44

#### 3.2.2 Independent component analysis

**Figure 2:**
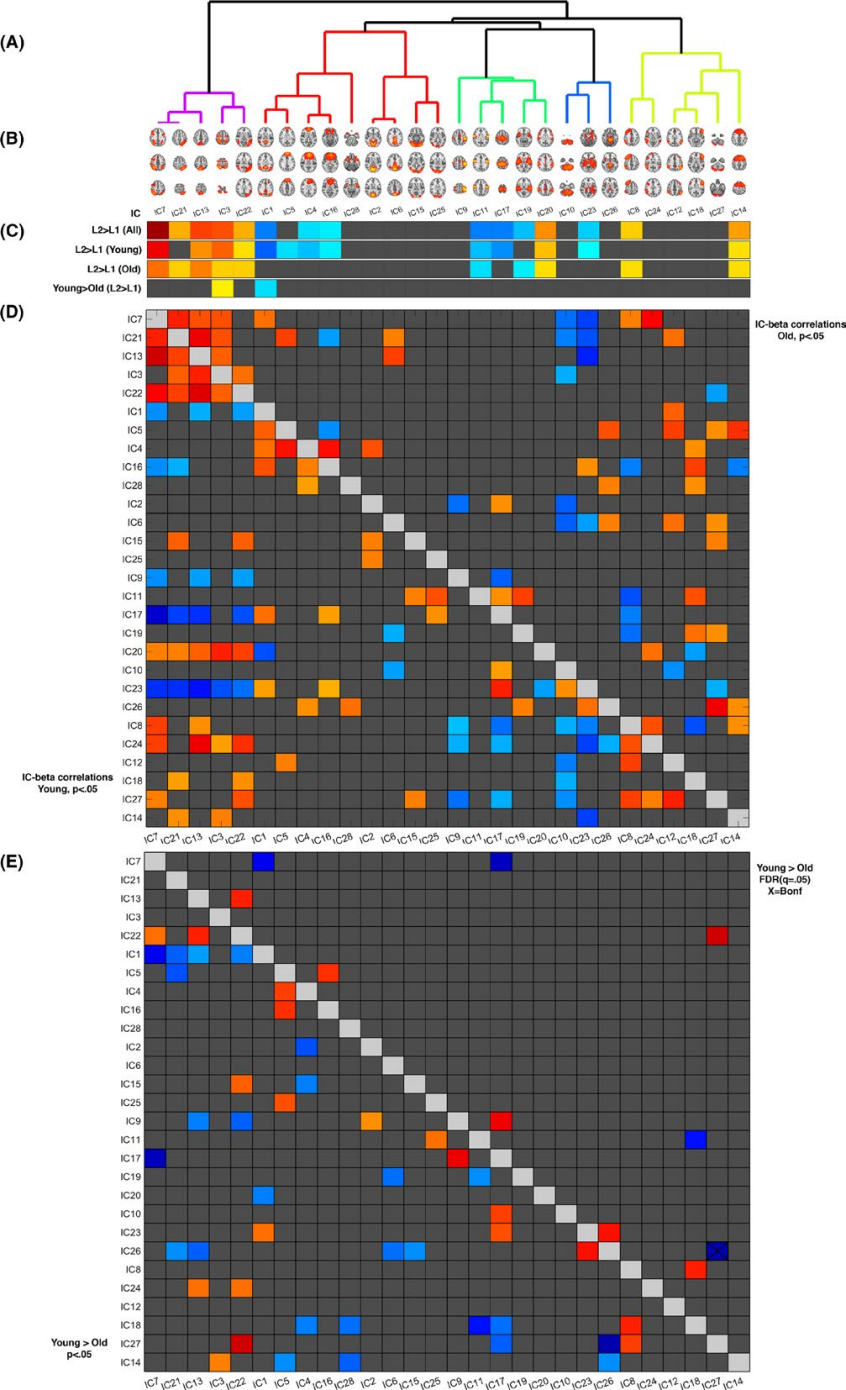
Panel A: Dendrogram showing clustering of nodes for the 28 components from the group ICA. Panel B: The 28 nodes from the group ICA. Panel C: GLM for all the independent components at the given contrasts. Panel D: The nominally significant (*p* < 0.5) beta correlations between the independent components, correlations in the old and young group are shown above and below the diagonal, respectively. Warm colors indicate high correlation, cold colors indicate low correlation. Panel E: Below the diagonal are the nominally (*p* < .05) significant differences in correlation (Young > Old). Above the diagonal are the FDR (*q* = 0.05) corrected differences in correlation (Young > Old). Warm colors indicate stronger correlations in the younger group, cold colors indicate stronger correlations in the older group. Squares marked with an X indicate the correlations surviving Bonferroni correction (378 independent tests)

From the 41 components generated by the group‐level ICA, 13 components were manually classified as noise components and discarded from further analysis, and the subsequent analyses were performed on the remaining 28 components (Fig. 2).

#### 3.2.3 Main effects of group and load and their interactions

Table 3 and Figure 2, panels A‐C summarize the estimated contrast parameters for the L2 > L1 contrast and the young > old contrast at L2 > L1 using the individual‐level GLMs and subject‐specific dual‐regression times series, for the 28 independent components generated from the group ICA. Briefly, hierarchical clustering grouped the components into five clusters, largely corresponding to task‐positive/DAN, DMN, somatosensory, brainstem/cerebellar, and frontoparietal clusters. The five strongest load effects were found for IC7 (DAN) (*t* = 12.21, *p* < .001), IC13 (posterior DAN) (*t* = 8.22, *p* < .001), IC 3 (DAN) (*t* = 7.83, *p *≤ .001), IC 1 (posterior DMN) (*t* = −7.02, *p* < .001), and IC11 (somatomotor) (*t* = −6.80, *p* < .001). The five strongest group effects at the L2 > L1 contrast were found for IC1 (posterior DMN) (*t* = −4.25, *p* < .001), IC3 (DAN) (*t* = 3.54, *p* = .001), IC 5 (anterior DMN) (*t* = −3.03, *p* = .004), IC 6 (posterior DMN) (*t* = −2.74, *p* = .009), and IC17 (somatomotor) (*t* = −2.70, *p* = .01), of which the two strongest (IC1 and IC3) remained significant after Bonferroni correction, indicating increased DAN activation and increased DMN deactivation in the young compared to the old group.

**Table 3:** Components sorted by main effect of load (L2 > L1) for all participants. Column 4 gives the *t*‐score for the young versus old contrast at L2 > L1. The rightmost column gives the respective brain networks/regions corresponding to each component. Values that are significant after Bonferroni correction for multiple comparisons (28 tests) are shown in bold

IC no.	L2 > L1, all participants, *t*‐score	*p*‐value	L2 > L1, Young>Old, *t*‐score	*p*‐value	Brain network/region
7	**12.21**	**<.001**	1.59	0.120	DAN
13	**8.22**	**<.001**	1.67	0.102	Post‐DAN
3	**7.83**	**<.001**	**3.54**	**0.001**	DAN
1	−**7.02**	**<.001**	−**4.25**	**<0.001**	Post‐DMN
11	−**6.80**	**<.001**	−0.50	0.620	Somatomotor
17	−**6.75**	**<.001**	−2.70	0.010	Somatomotor
20	**5.98**	**<.001**	2.13	0.039	Supramarginal gyrus
14	**5.78**	**<.001**	−0.98	0.332	Superior frontal gyrus
22	**5.30**	**<.001**	1.68	0.100	Lateral occipital
21	**5.11**	**<.001**	0.09	0.932	Left lateralizedDAN
19	−**5.11**	**<.001**	−0.35	0.729	Auditory/temporal
23	−**4.84**	**<.001**	−1.27	0.211	Subcortical
4	−**4.54**	**<.001**	−2.25	0.030	Ant. DMN
8	**4.49**	**<.001**	−0.76	0.450	Right frontoparietal
16	−**4.06**	**<.001**	−0.67	0.504	Ant. DMN
12	−3.01	.004	−2.29	0.027	Paracingulate
26	2.98	.005	−2.01	0.051	Brainstem
15	2.97	.005	0.10	0.922	Visual
28	−2.30	.026	−0.78	0.440	Insula
5	−2.29	.027	−3.03	0.004	Ant. DMN
9	−1.80	.079	−1.27	0.210	Somatomotor
18	−1.79	.080	−0.38	0.704	Left inferior frontal
25	−1.44	.156	−0.10	0.919	Visual
10	1.42	.163	0.48	0.634	Cerebellum
24	1.23	.226	0.09	0.927	Angular gyrus
27	−0.86	.395	−2.49	0.017	Cerebellum
2	0.44	.663	−1.78	0.083	Visual
6	0.28	.784	−2.74	0.009	Post‐DMN

#### 3.2.4 DAN and DMN activations and associations between these networks

**Figure 3:**
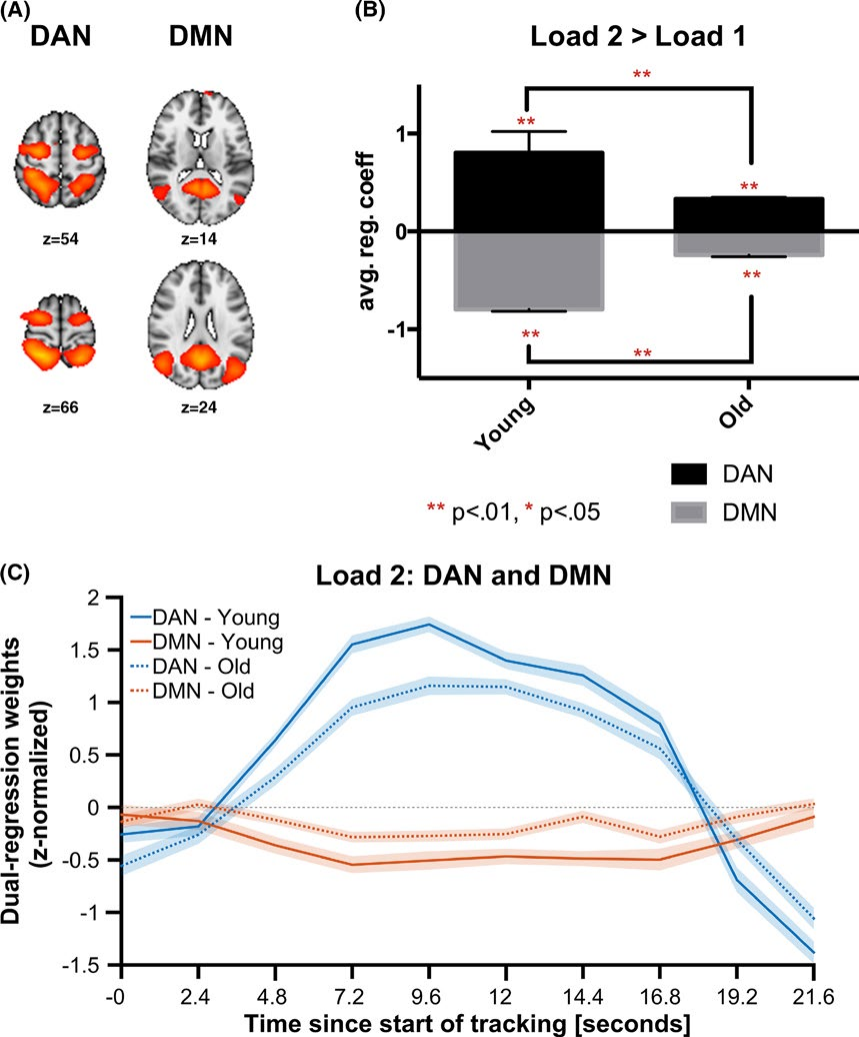
ICA: (A) Spatial maps with selected components representing DAN (right) and DMN (left), (B) Average differences in beta estimates between L2 and L1 for the two networks, (C) Time series for DAN and DMN in both groups during L2

Figure 3A shows the two components showing significant group differences, representing the DMN (IC1) and the DAN (IC3), respectively. Figure 3C shows the average blocked time‐series for the two components during L2. Table 4 summarizes the regression coefficients (betas) for each component for each of the load conditions.

**Table 4:** Beta coefficients reflecting the component time series model fit with the task design. Bold: Significantly (*p* < .05) different from zero as indicated by one sample *t*‐tests. Standard deviation is given in the parentheses

	Young		Old	
	L1	L2	L1	L2
DAN	**1.89 (0.44)**	**2.70 (0.77)**	**1.47 (0.45)**	**1.80 (0.51)**
DMN	0.15 (0.45)	−**0.65 (0.50)**	−0.12 (0.51)	−**0.36 (0.33)**

A repeated‐measures ANOVA with load as within subject factor and group as between subject factor revealed significant main effects of Load [*F*(1,43) = 72.97, *p* = 8.21E‐11, partial η^2 ^= .63] and Group [*F*(1,43) = 18.1, *p* = . 0001, partial η^2 ^= .296], and a significant Load by Group interaction [*F*(1,43) = 12.55, *p* = .001, partial η^2 ^= .226] on DAN activation. Post hoc group comparisons within the load conditions, using an ANCOVA with gender and group as fixed factors, revealed significant differences in DAN L1 [*t*(42) = 3.35, *p* = .002] and DAN L2 [*t*(42) = 5.13, *p* = 7.0E‐7], indicating stronger DAN activations in the young group compared to the old, in both load conditions.

A repeated‐measures ANOVA revealed a significant main effect of Load [*F*(1,43) = 63.46, *p* = 5.29E‐10, partial η^2 ^= .60], no significant main effect of Group [*F* = (.005), *p* = .946, partial η^2^ = .00011], and a significant Load by Group interaction effect [*F*(1,43) = 18.05, *p* = .00011, partial η2 = .296] on DMN deactivation. ANCOVA testing for group differences within the two load conditions in DMN activations revealed significant differences in DMN L1 [*t*(42) = 2.22, *p* = .032] as well as DMN L2 [*t*(42) = −2.30, *p* = .027], indicating increased and decreased DMN deactivation in the old group in the L1 and L2 condition, respectively.

Figure 3B summarizes the average difference between the L1 and L2 regression coefficients for the two groups. Paired samples t‐tests revealed greater load‐dependent DAN activations both in the young [mean difference = .804, *SD *= (.521), *t*(23) = 7.55, *p* = 1.15E‐7] and the old [mean difference = .332, *SD *= .336), *t*(19) = 4.53, *p* = .00005] group. Further, both the young [mean difference = −.796, *SE *= .489), *t*(23) = −7.97, *p* = 4.58E‐8] and the old (mean difference = −.242, *SE* .367, *t*(19) = −3.04, *p* = .007) group showed greater DMN deactivations during L2 compared to L1.

ANCOVA revealed a significant [*t*(42) = 4.01, *p* = .00025] effect of group on the difference in DAN activation between L2 and L1, indicating larger load‐dependent activations in the young compared to the old group. In addition, ANCOVA revealed a significant [*t*(42) = −4.27, *p* = .00011] effect of group on the difference in DMN deactivation between L2 and L1, indicating stronger load‐dependent deactivations in the young compared to the old group.

ANCOVA testing for associations between betas for the DAN and the DMN within load conditions revealed no significant associations for either group during L1 [young: *t*(23) = .42, *p* = .678; old: *t*(19) = 1.81, *p* = .088] or L2 [young: *t*(23) = .27, *p* = .792; old: *t*(19) = 1.17, *p* = .260). For the difference between L2 and L1, the ANCOVA revealed a significant association between DAN and DMN activation for the old group [*t*(19) = 2.18, *p* = .042], but not for the young [*t*(23) = −.65, *p* = .526], indicating a positive association between DAN and DMN activation when increasing load from L1 to L2 in the old group.

### 3.3 Associations between component activation

We used the beta coefficients for all the 28 independent components generated from the group ICA to test for associations between component activation in both groups for the L2 > L1 contrast (Fig. 3, panel D). In a graph theoretical framework, the brain is modeled as a network that can be graphically represented by an assembly of nodes and edges. Here, the nodes represent the respective components and edges correspond to the temporal correlations between the said components. Across groups, positive correlations were primarily found between task‐positive components within the DAN. Negative correlations were primarily found between subcortical and task‐positive DAN components and between somatomotor and task‐positive DAN components. Component correlations within age groups revealed a higher number of correlated and anticorrelated components in the young compared to the old group; 92 out of 378 edges showed significant correlations at the nominal level in the young group; for the old group, 64 out of 378 edges were found nominally significant. Strongest positive correlations between components in the young group were found between IC7 (DAN) and IC13 (posterior DAN) (*r* = .82, *p* = 1.22E‐6), IC13 (posterior DAN) and IC22 (lateral occipital) (*r* = .79, *p* = 4.35E‐6), and between IC13 (posterior DAN) and IC24 (angular gyrus), (*r* = .76, *p* = 1.63E‐5). Strongest negative correlations between components in the young group were found between IC7 (DAN) and IC17 (somatomotor) (*r* = −.87, *p* = 3.88E‐8), IC13 (posterior DAN) and IC23 (subcortical) (*r* = −.73, *p* = 4.36E‐5) and between IC13 (posterior DAN) and IC17 (somatomotor) (*r* = −.68, *p* = 2.31E‐4). Strongest positive correlations between components in the old group were found between IC13 (posterior DAN) and IC21 (left lateralized DAN) (*r* = .77, *p* = 5.16E‐5), IC26 (brainstem) and IC27 (cerebellum) (*r* = .76, *p* = 6.17E‐5) and between IC7 (DAN) and IC24 (angular gyrus) (*r* = .72, *p* = 2.22E‐4). Strongest negative correlations between components in the old group were found between IC13 (posterior DAN) and IC23 (subcortical) (*r* = −.70, *p* = 3.74E‐4), IC7 (DAN) and IC23 (subcortical) (*r* = −.63, *p* = 2.30E‐3) and between IC8 (right frontoparietal) and IC 18 (left inferior frontal) (*r* = −.63, *p* = 2.30E‐3).

Figure 3E summarizes the age‐related differences in correlations between the 28 independent components at nominal, FDR, and Bonferroni‐corrected levels of significance for the L2 > L1 contrast, and Table 5 lists the edges with respective correlations coefficients within both groups and Fisher's z‐scores comparing group differences. Using the more conservative Bonferroni correction, only one edge was significantly stronger in the older group, namely IC26 (brainstem)‐IC27 (cerebellum), and no edges were stronger in the younger group. Briefly, using FDR correction, stronger correlations were found between two DAN components (IC13–IC22) showing increased load‐dependent activations (task positive) and within task‐negative DMN components (IC5–IC16), in the young group. Stronger correlations were found between IC7 (DAN) and IC1 (posterior DMN), as well as between IC7 (DAN) and somatomotor IC17 (somatomotor), in the older group. Edges IC15 (visual)‐IC18 (left inferior frontal) and IC22 (lateral occipital)‐IC27 (cerebellum) were at a nominal significance level (*p* < .05, uncorrected) positively correlated in the young group and negatively correlated in the old group. The edge IC1 (posterior DMN)‐IC7 (DAN) was negatively correlated in the young group, but positively correlated in the old group.

**Table 5:** The 13 edges that showed FDR‐corrected significant differences in correlations between the young and the old group for the L2 > L1 contrast, corresponding to Figure 2, panel E, above the diagonal. Columns to the left show the correlation coefficients and corresponding *p*‐values within the young and the old group, respectively. The two rightmost columns give the Fisher's r‐to‐z values between the two groups' correlations coefficients with corresponding *p*‐values. Bold indicates nominally significant component correlation within the groups

Edge	Young, *r*	Young, *p*	Old, *r*	Old, *p*	Fisher's *Z*	*p*
IC1–IC7	−.49	**0.01**	.50	**0.02**	−3.41	6.43E‐4
IC7–IC17	−.87	**3.38E‐3**	−.11	0.65	−3.79	1.50E‐4
IC13–IC22	.79	**4.35E‐6**	.16	0.49	2.84	4.51E‐3
IC5–IC16	.35	0.09	−.48	**0.03**	2.78	5.40E‐3
IC9–IC17	.36	0.09	−.59	**4.99E‐3**	3.26	1.11E‐3
IC23–IC26	.52	**8.88E‐3**	−.36	0.11	2.96	3.05E‐3
IC11–IC18	−.33	0.11	.58	**6.04E‐3**	−3.12	1.79E‐3
IC8–IC18	.18	0.40	−.62	**2.55E‐3**	2.84	4.47E‐3
IC15–IC18	.59	**2.57E‐3**	−.77	**3.87E‐5**	5.30	1.15E‐7
I22–IC27	.56	**4.14E‐3**	−.45	**0.04**	3.49	4.84E‐4
IC26–IC27	−.24	0.26	.76	**6.17E‐5**	−3.88	1.06E‐4

#### 3.3.1 Associations with task performance

Two components corresponding to the DAN and the DMN showed significant load by group interaction effects. Based on these effects and a wealth of literature implicating these two networks as imperative to visual attention performance, the beta coefficients for the DAN and DMN were tested for correlations with task performance as indexed by the average percentage of correct responses for the two groups, respectively.

ANCOVAs assessing the relationship between the estimated betas (L2 and L2‐L1) for DAN and DMN, and MOT tracking accuracy revealed for DAN L2, no main effect of group [*F*(1,40) = .15, *p* = .700, partial η^2 ^= .004], or DAN L2 activation [*F*(1,40) = 2.20, *p* = .146, partial η^2 ^= .052], and no interaction between group and DAN L2 activation [*F*(1,40) = .48, *p* = .492, partial η^2 ^= .012]. For DMN L2, there was a main effect of group [*F*(1,40) = 4.48, *p* = .038, partial η^2 ^= .103], but no main effect of DMN L2 [*F*(1,40) = .30, *p* = .585, partial η^2 ^= .008], and no interaction effect [*F*(1,40 = .32, *p* = .572, partial η^2 ^= .008]. No significant relationship was revealed between MOT tracking accuracy and the relative difference in load‐dependent activations for either network (DAN and DMN L2‐L1).

#### 3.3.2 Motion correction

The dataset yielded significantly more in‐scanner motion in the old group [(*df*)*t* = −3.9, *p* < .0001], and FIX removed a significantly higher number of components from the older group, *t*(19) = −2.2, *p* < .05, and also removed more of the variance from the raw fMRI data, both in terms of absolute and relative variance, both *t*(19) = −2.8, *p* < .05), which were significantly correlated with amount of subject motion across groups (*r* = .52, *p* < .0001).

In order to rule out that any group differences were induced by the cleaning procedure, we estimated load responses on the uncleaned dataset. The effects reported remained largely unchanged.

## 4 Discussion

Aging is associated with a manifold of changes in the brain structure and function resulting in altered behavioral performance and decline in cognitive faculties. Using a combination of fMRI‐based voxel and multivariate analyses across a range of large‐scale brain networks obtained during multiple object tracking, we have demonstrated age‐related differences in tracking performance and associated brain network recruitment, in particular related to task‐related activation and deactivations of the DAN and DMN, respectively. Compared to the young participants, the older subjects showed reduced tracking accuracy. Voxel and brain network‐level fMRI analyses converged on decreased load‐dependent activation of the DAN, and decreased load‐related deactivations of the DMN during tracking in the old group, suggesting differential age‐related alterations in task‐positive and task‐negative brain networks. Investigating the age‐ and load‐dependent effects in all of the 28 components generated from the group ICA, we found a significant group by load interaction in two components representing the DAN and the DMN, respectively. This suggests that these two brain networks are engaged during the MOT task as well as sensitive to aging effects.

Furthermore, we have demonstrated significantly stronger correlations between the levels of activation in components within both the DAN and the DMN for younger adults. Conversely, task‐positive DAN and DMN components were positively correlated in older adults and negatively correlated in younger adults.

### 4.1 Effects of task and age on task‐positive networks

Studies investigating the brain network involvement in visual attention consistently report task‐related activations in the DAN (Corbetta & Shulman, 2002; Kastner & Ungerleider, 2000). This frontoparietal network is hypothesized to be involved in the goal‐directed (top‐down), endogenous component of attentional processing as opposed to the exogenous, stimulus‐driven (bottom‐up) component (Shulman et al., 1997). Less consistent findings have been reported when studying the age‐related effects on DAN activation; some studies have found a relative increase in activation for older adults, possibly reflecting functional compensation for inefficient bottom‐up processing (Cabeza et al., 2004; Madden, 2007). However, a longitudinal study by Nyberg et al. (2010) reported age‐related reduced activations in the same areas, complicating a straight‐forward interpretation of the DAN in relation to neurocognitive aging.

With diverging literature in mind, we hypothesized an altered DAN response in older relative to younger adults without making strong predictions with regard to the specifics of these alterations. We observed strong DAN activation in both age groups when subjects were engaged in the task, and increasing load demand from one to two targets resulted in a further increased DAN activation. Group comparison revealed significantly stronger DAN activation for younger relative to older adults, and this activation pattern amplified with increasing task demand. Furthermore, the relative increase in DAN activation observed with increasing load demand was significantly greater for younger adults, possibly representing a greater capacity for DAN recruitment in response to increased attentional demand for younger adults. Thus, results obtained from this study support an age‐related diminished activation pattern for the DAN along with less capability for load‐dependent activation increase for older adults.

### 4.2 Effects of task and age on task‐negative networks

Mediating introspection and self‐referential thought, the DMN represents an antagonistic function to that of the task‐driven DAN. Also known as the task‐negative network, the DMN is deactivated in response to tasks involving selective visual attention reflecting a reallocation of resources from monitoring of the self and the environment to external and goal‐oriented behavior. Although the DMN is not task‐negative *per se* in that the DMN is indeed engaged in active cognitive processes requiring internal focusing (Golland et al., 2007), the value in dichotomizing these two networks stems from studies where attention demanding tasks engage the DAN and passive fixation relative to task reliably engages the DMN (Raichle et al., 2001; Shulman et al., 1997). Our results are in line with the existing literature; we observed strong task‐related DMN deactivations for both groups with diminished deactivations for the older relative to the younger group. Similar (although with opposite direction) to the activation pattern observed for the DAN—increasing task demand resulted in stronger DMN deactivations for both groups with the younger group having a significantly stronger load‐dependent response compared to the older group.

These findings are in line with several previous observations (Grady et al., 2006; Persson et al., 2007), and suggest that reduced DMN modulation with increasing age can negatively affect attentional processing by increasing vulnerability to irrelevant distractions.

The ICA approach we employed in this study parsed the DMN into five components, the DMN component (IC 1) showing load and group interaction effects and that was included in the subsequent analyses, corresponded anatomically to the posterior DMN (precuneus and angular gyrus). Our findings fall in line with a number of studies in healthy aging and Alzheimer's disease, that have observed the posterior DMN to have an increased susceptibility to decreases in task‐induced deactivations (Hafkemeijer, van der Grond, & Rombouts, 2012; Mevel, Chételat, Eustache, & Desgranges, 2011). The same areas are also particularly vulnerable to amyloid deposits (Sperling et al., 2009), a proposed cause of functional disruption and aberrant network activity, even in clinically healthy subjects (Sperling et al., 2010).

### 4.3 Association between brain network activation

Based on the interlinked and dynamic relationship between various brain networks during the execution of cognitive tasks, in particular between the DAN and DMN, a critical aim of this study was to investigate the associations between the levels of activation of different brain networks and how their relationship is affected in aging. Importantly, if the level of task‐related activation in one network (e.g., the DAN) is highly correlated with the level of activation in another network (e.g., the DMN), this may indicate that the activation levels of the two networks are reflecting partly overlapping mechanisms. We found no such significant association between two selected components representing the DAN and the DMN at the L1 or the L2 load conditions for either of the age groups, a finding that could suggest that the mechanisms of brain network level activation in cognitive aging, for these two networks are relatively independent. However, this interpretation might be an oversimplification of the complex network dynamics underlying attentional processing. Considering the engagement of the DMN in goal‐directed internally focused tasks such as autobiographical planning, recent studies (Di & Biswal, 2014; Spreng, Stevens, Chamberlain, Gilmore, & Schacter, 2010) have elucidated the confounding role of an anatomically interposed frontoparietal executive control network and its function in flexibly coupling with the DAN or DMN when engaged in external or internal oriented tasks, respectively. Further, investigating aging effects, Spreng and Schacter (2012) found for older compared to younger adults a relative inability to decouple the control network from the default network during a visuospatial task. The researchers attributed the failure of DMN deactivation not to intrinsic DMN dysfunction, but rather reduced network flexibility and range of dynamic network modulation in response to different task demands. The magnitude of load‐dependent activations (L2‐L1) in a range of components was significantly correlated across groups. In particular, we found strong positive correlations within task‐positive components, between visual and task‐positive components and within two DMN components, indicating that subjects with strong load‐dependent increase in activation in one component also showed strong load‐dependent activation in the other components and subjects with strong load‐dependent decrease in activation in one component also showed load‐dependent deactivation in another component. Similarly, we observed strong negative correlations between task‐positive and task‐negative components and between subcortical and task‐positive components, indicating the opposite relationship. The level of co‐activation across components pertains to the system‐level coordination of brain networks during cognitive processing, and comparing the correlation between groups yields a window into the age‐related differences in this brain network coordination. Interestingly, we identified significantly stronger correlations within DAN as well as within DMN components in the younger group and conversely, stronger correlations between DAN and DMN components in the older group, indicating a load‐dependent response shifting from increased within‐network specificity in younger adults to a more between‐network dependence in older adults. Disrupted functional connectivity with advancing age has been proposed to reflect a reduction in specialization and segregation of brain systems (Chan et al., 2014). This brain dedifferentiation and reduction in diversity (Ferreira et al., 2015) may reduce the flexibility and dynamic repertoire of large‐scale brain networks, which in turn contribute to age‐related cognitive decline (Chou, Chen, & Madden, 2013). Along with the robust group effects on the load‐dependent activation of the DMN and DAN, these results suggest altered coordination of brain networks during cognitive processing in aging.

### 4.4 Associations between task performance and DAN and DMN activation

As expected, we observed a significant group difference in MOT performance accuracy between younger and older adults, as well as a significant effect of load, but no interactions.

Investigating the relationship between MOT accuracy and component beta estimates (DAN and DMN L2 and L2‐L1), we found for DMN L2, a significant main effect of age group, but no significant effect of DMN deactivation and no interaction effect on performance accuracy. No significant associations were found for DMN L2‐L1, DAN L2, or DAN L2‐L1. The weak relationship between activation levels and performance accuracy could be due to the relatively low task demand. Investigating age differences in the MOT task, Sekuler, McLaughlin, and Yotsumoto (2008) demonstrated that younger adults were able to track up to four target objects simultaneously while older adults managed to track only three. Our implementation of the MOT task was limited to tracking a maximum of two target objects to ensure that participants of both groups were able to maintain task‐focus throughout each trial. However, this restriction prevented participants from achieving maximal attentional load demand, allowing for ceiling effects and limited our capacity to make any strong inferences about how specific network properties relate to task performance at high load.

### 4.5 Limitations

This study does not come without limitations. Head movement is a ubiquitous concern in studies of network properties, and we found significantly more in‐scanner motion in the old group. We used a sensitive approach for denoising of fMRI data by automated classification of ICA components on an individual level (FIX; Salimi‐Khorshidi et al., 2014). Although there inevitably will be an effect of motion in any given fMRI experiment, using such a validated approach (Pruim et al., 2015) minimizes noise contamination. However, since the cleaning procedure removed more variance from the old participants, we estimated both DAN and DMN load‐responses on the uncleaned datasets in order to rule out that any age differences was induced by the cleaning procedure. The effects reported remained largely unchanged.

Since we included healthy subject on both ends of the adult age spectrum, we do not have data covering a continuous age range. Therefore, our data cannot determine whether changes in network patterns follow a linear or nonlinear curve or if there is a critical age at which a cut‐off point is reached and dramatic decline in attentional ability is observed. Full‐scale IQ observed for both groups were above average. Considering that the sample was not drawn randomly from the population, but rather based on convenience—this was not an unexpected finding, yet it does influence generalizability. A study by Dixon et al. (2004) investigating episodic memory retrieval found a similar, gradual age‐related decline in an advantaged convenience sample and a low‐education population‐based sample, suggesting that although population‐based samples are more representative, the same pattern of age‐related changes in higher cortical functions is retained in convenience samples.

As previously addressed, the restriction to two MOT load conditions was done to ensure that the participants indeed were engaged in the MOT task and that neuronal activity more accurately reflected attentional effort and not mind wandering due to loss of focus. For some of the participants, especially in the young group, the task demand might have been insufficient to elicit a strong network response, and we cannot exclude that including higher load conditions would have revealed stronger group effects and possibly group by load interactions. However, using tracking accuracy as an index of attentional performance, this design proved sensitive to group and load effects validating its use within the scope of our investigation.

## 5 Conclusion

Using MOT as means of measuring visual attention, our results further support a substantial amount of research reporting an age‐related attentional decline. fMRI analysis including a range of large‐scale brain networks revealed age‐related alterations in network recruitment during mental tracking consisting of diminished activations of the DAN and diminished deactivations of the DMN in older relative to younger participants. Although these brain network level reductions may reflect a general signature of the aged brain, we did not observe any robust associations with task performance in the older group, possibly due to the relatively low task demands. Lastly, we identified several robust correlations in brain network activations, and also significant group differences in a subsample of these brain network activation correlations; we found stronger correlations within DMN and within DAN components for younger adults and stronger correlations between DAN and DMN components for older adults, indicating age‐related alterations in the coordinated network‐level activation during attentional processing. The correlation between DAN and DMN activation was low, suggesting that while some dependencies indeed exist between several of the estimated brain networks, the age‐related alterations in DAN and DMN responses to attentional demands may reflect independent mechanisms.

## Funding Information

This work was funded by the Research Council of Norway (204966/F20), the South‐Eastern Norway Regional Health Authority (2013054, 2014097, 2015073), and the Norwegian Extra Foundation for Health and Rehabilitation (2015/FO5146).

## Conflict of Interests

None declared.
