# Supplementary figures and images for: Age‐related differences in brain network activation and co‐activation during multiple object tracking
Source: Brain Behav. 2016 Sep 7;6(11):e00533. doi: 10.1002/brb3.533 (PMC5102637; doi:10.1002/brb3.533)

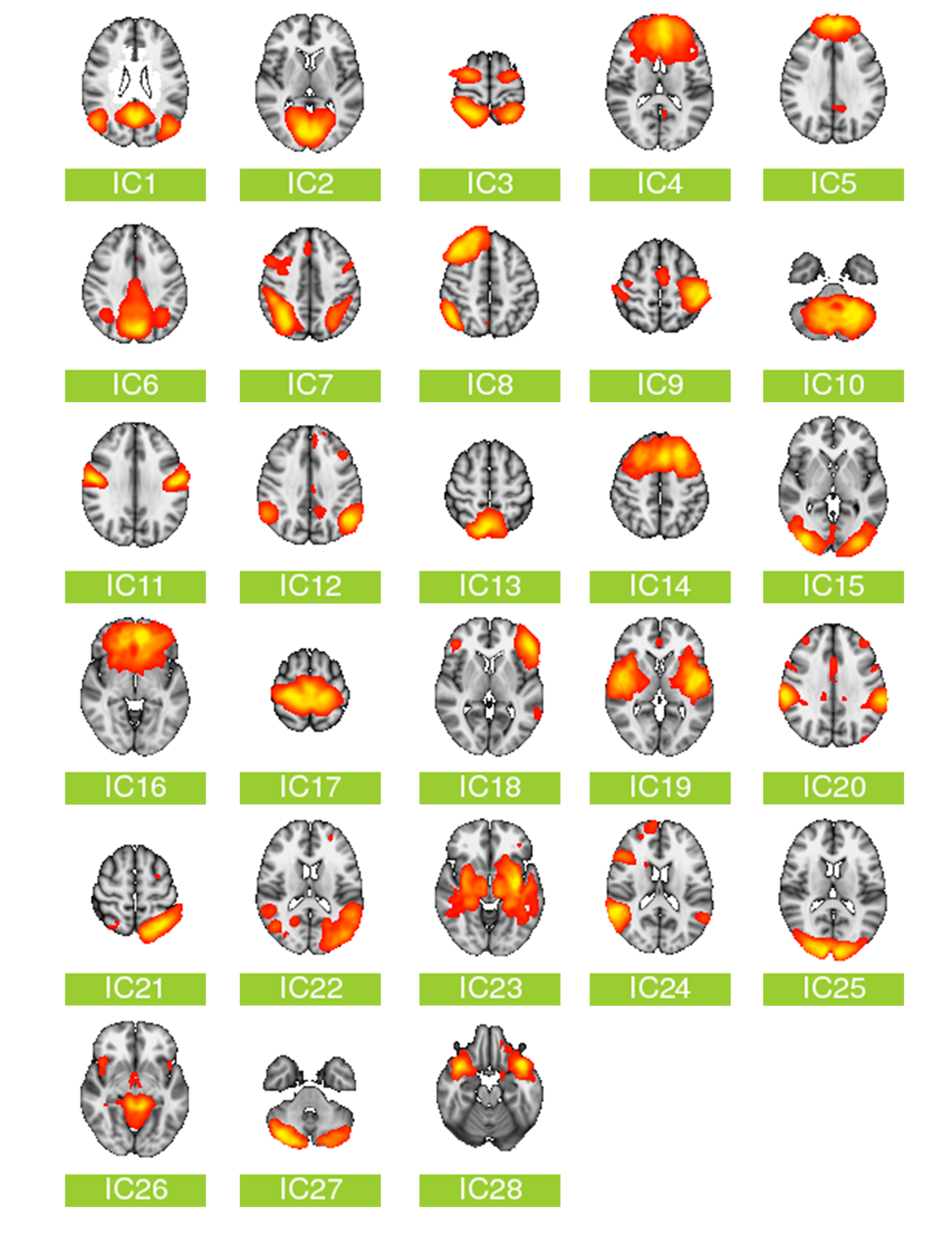

Supplement: Supplementary file 1 [file BRB3-6-e00533-s001.tif]
